# Identity on trial: legitimacy-focused invalidation and social anxiety in bisexual and nonbinary people

**DOI:** 10.3389/fpsyg.2026.1800884

**Published:** 2026-04-09

**Authors:** Daniela Fumega, Martin Stork

**Affiliations:** CIPsi – Centro de Investigação em Psicologia, Escola de Psicologia, Universidade do Minho, Braga, Portugal

**Keywords:** bisexual, identity invalidation, minority stress, misgendering, nonbinary, rejection sensitivity, safety behavior, social anxiety

## Introduction

Minority stress models explain elevated mental health burden in LGBTQ+ populations by emphasizing cumulative stigma-related experiences (e.g., discrimination, victimization) and proximal processes such as expectations of rejection, concealment, and internalized stigma (Hatzenbuehler, 2009; Meyer, 2003). Microaggressions are one common interactional form these experiences can take (Nadal et al., 2011; Nadal, 2019). Recent subgroup research also suggests that social anxiety may be especially elevated in bisexual+ and transgender/nonbinary groups (Mahon et al., 2023; Wadsworth and Hayes-Skelton, 2015). Yet, minority stress is often treated as a broad risk factor for “distress,” which can obscure which common interactional experiences most directly recruit specific psychological mechanisms. This matters for social anxiety because it is maintained by identifiable cognitive-behavioral processes that can be measured and targeted.

We argue that identity invalidation—being treated as “not really” one's stated sexual or gender identity—deserves focused attention for social anxiety in bisexual+ (bi+; i.e., bisexual, pansexual, and other non-monosexual identities) and nonbinary/gender-diverse people. This focus may be especially relevant given evidence that social anxiety is not distributed uniformly across sexual- and gender-identity subgroups. These identities are often stereotyped as ambiguous, transitional, or incoherent, making social recognition relatively fragile and routinely contested. However, invalidation may operate somewhat differently across groups. For bi+ people, invalidation often involves erasure and legitimacy challenges (e.g., “pick a side,” relabeling based on a current partner, gatekeeping about being “queer enough”) (Feinstein et al., 2019), often in ways tied more to disclosure, relationship context, or others' interpretations of identity. For nonbinary and other gender-diverse people, invalidation often involves misrecognition and binary enforcement (Johnson et al., 2020, 2024), including misgendering (McLemore, 2015), and may arise as a more immediate feature of everyday interactions through pronouns, names, forms of address, or judgments linked to gender expression (Arijs et al., 2023).

Social anxiety is centrally organized around perceived social evaluation and is maintained by anticipatory threat, self-focused attention, safety behaviors, and pre- and post-event processing (Clark and Wells, 1995). More broadly, cognitive-behavioral models emphasize biased processing of social-evaluative information (Rapee and Heimberg, 1997). We propose that identity invalidation repeatedly recruits these same processes, but with a critical distinction from prototypical presentations of social anxiety disorder: in many settings the social threat is more realistic. When legitimacy is questioned, negative outcomes (loss of belonging, conflict, reduced access, safety concerns) may be more likely and more consequential. The point, then, is not simply exaggerated threat, but how repeated legitimacy threat may foster rigid self-monitoring, safety behaviors, and broader anticipatory anxiety over time. Minority stress helps explain why the threat exists; a social-anxiety framework helps explain how fear and avoidance may become maintained.

In this Opinion, we conceptualize identity invalidation as a recurring legitimacy-focused social-evaluative trigger and outline a concise maintenance loop. The contribution is mechanism specificity: we map identity invalidation onto established social-anxiety maintenance processes, describe context-calibrated clinical targets that do not deny real-world risk, and propose measures and study designs to test the model.

## Identity invalidation as social-evaluative minority stress

We define identity invalidation as recurrent misrecognition, erasure, or gatekeeping in which others deny or refuse to accept a person's stated identity, or force them into ill-fitting categories (Feinstein et al., 2019; Johnson et al., 2020, 2024). It overlaps with sexual-orientation and gender-identity microaggressions and is often captured in microaggression frameworks, but we treat it as a legitimacy-focused subset of interactional minority stress (Nadal et al., 2011; Nadal, 2019). Invalidation can be discrete (e.g., misgendering; “you're not really bi+”) or occur in predictable contexts where identity is assumed or policed (e.g., pronouns assumed; binary forms) (Feinstein et al., 2019; Johnson et al., 2020; McLemore, 2015).

Two features make invalidation especially relevant for social anxiety. First, it is often mundane and recurrent in everyday interactions (Feinstein et al., 2019; Johnson et al., 2020; Nadal et al., 2011). Second, it targets legitimacy in the moment —“Do you belong here as who you say you are?”—and qualitative accounts describe rumination and self-doubt afterward, suggesting a direct route into maintenance processes (Feinstein et al., 2019; Johnson et al., 2020, 2024).

Invalidation varies in intensity and impact. Subtle forms include doubt-implying “clarifications,” partner-based relabeling, or treating bisexual+ or nonbinary identity as a phase (Feinstein et al., 2019; Johnson et al., 2020). More overt forms include explicit legitimacy challenges or refusal to use affirmed names/pronouns (Johnson et al., 2020; McLemore, 2015). At the severe end are hostile, coercive, or violent experiences—and chronic, intense invalidation—that can be conceptualized as traumatic invalidation (Ballard, 2024; Cardona et al., 2022). Across this continuum, repeated exposure may strengthen anticipatory threat and promote scrutiny-minimizing identity-management (e.g., concealment, avoidance, self-silencing), while some report approach-oriented coping (e.g., advocacy, help-seeking) (Ballard, 2024; Maiolatesi et al., 2023; Pachankis, 2007).

Invalidation is embedded in the broader minority stress system (Hatzenbuehler, 2009; Meyer, 2003). It can heighten stigma-specific rejection sensitivity, linked to social anxiety outcomes in sexual minority samples (Maiolatesi et al., 2023), and promote concealment and related strategies that reduce immediate scrutiny but can maintain fear when rigid or generalized (Pachankis, 2007). Invalidation may also amplify shame and post-event processing after interactions, interfacing with core maintenance processes in cognitive models of social anxiety (Cardona et al., 2022; Clark and Wells, 1995).

## A maintenance loop for social anxiety

**Figure 1:**
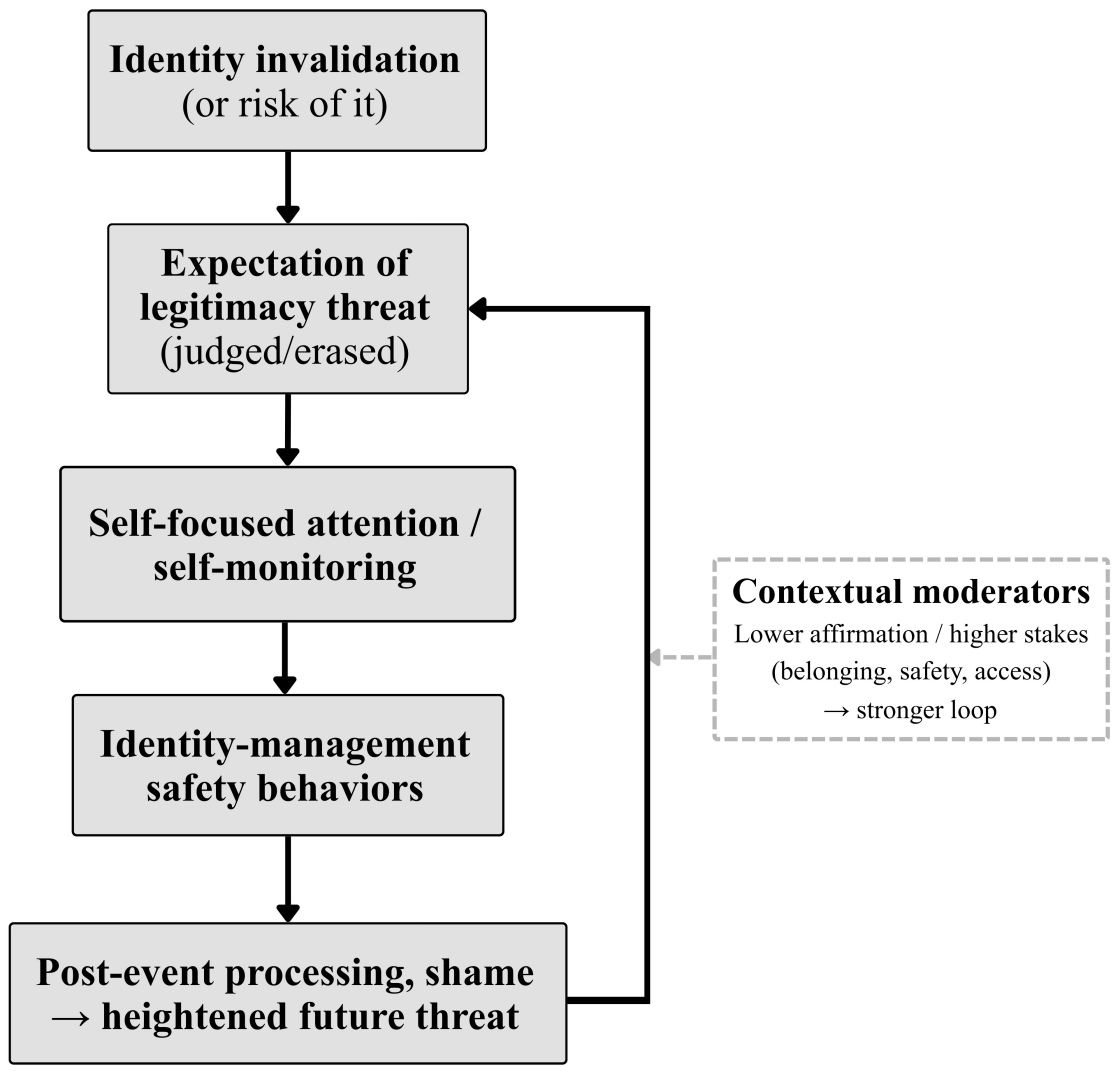
Identity invalidation–social anxiety maintenance loop. Identity invalidation (or risk of it) increases legitimacy-threat expectations, which increase self-focused attention and identity-management safety behaviors. These responses increase post-event processing/shame, heighten future threat, and feed back into threat expectations. Lower affirmation/higher stakes are proposed to strengthen the loop.

We propose a concise loop that may be especially relevant when repeated invalidation shifts from context-specific vigilance to broader anticipatory anxiety: identity invalidation (or its perceived risk) increases legitimacy-threat expectations (being judged, misrecognized, or erased), which increase self-focused attention and identity-management safety behaviors. These responses increase post-event processing (rumination, self-criticism, shame), heighten future threat, and feed back into legitimacy-threat expectations (Figure 1). The loop should be strongest when invalidation is socially consequential (belonging, safety, access) and coping relies on monitoring/avoidance rather than flexible responding. The broad loop may be similar across groups, but the cues that trigger it and the most common identity-management strategies may differ (e.g., correction/pronoun management vs. concealment/selective disclosure).

### Invalidation (or risk) → legitimacy threat expectations

Repeated invalidation can raise both the perceived likelihood and cost of legitimacy-related negative evaluation (e.g., “I'll be seen as incoherent,” “I'll lose belonging”). Expectations can be triggered by policing cues (e.g., binary forms; groups with known invalidators) and may include vigilance about being misclassified, carried in part by stigma-specific rejection sensitivity and concealment-related threat processes (Maiolatesi et al., 2023; Pachankis, 2007).

### Threat expectations → self-focused attention

As in established models, perceived evaluation shifts attention inward (Clark and Wells, 1995; Rapee and Heimberg, 1997). Under invalidation-related threat, monitoring targets identity cues and impression management (how one is read as “legitimate”), whether through gendered interactional cues or interpretations of disclosed identity, increasing cognitive load and reducing spontaneous engagement.

### Self-focused attention → identity-management safety behaviors

Strategies can function as safety behaviors when their primary aim is to reduce scrutiny: concealment, softening labels, avoiding correction, appeasing, pre-scripting, or over-explaining, with some strategies likely more common in certain invalidation contexts than others. They may be adaptive in unsafe settings, but become maintaining when generalized to relatively safe or ambiguous contexts: they reduce short-term threat while limiting corrective experiences and reinforcing the rule that safety depends on constant management (Clark and Wells, 1995; Pachankis, 2007). Concealment is also linked to social phobia symptoms in sexual minority young adults (Cohen et al., 2016).

### Safety behaviors → post-event processing → future threat

Post-event processing (replaying, self-criticism, shame) can strengthen future threat expectations (Clark and Wells, 1995). Rumination is prominent in daily life among transgender and gender diverse people, including themes involving safety concerns, interpersonal rejection, and gender as experienced through self and others (Puckett et al., 2022). Prospectively, shame contributes to sexual-orientation disparities in social anxiety and mediates links between interpersonal stigma (e.g., family rejection, childhood bullying) and later social anxiety, supporting shame as a feed-forward mechanism in this loop (Pachankis et al., 2024).

## What is distinctive about this pathway?

A key boundary condition is realism. In many cases of social anxiety disorder, threat appraisals are biased upward: people may overestimate the likelihood or costs of negative evaluation and underestimate their ability to cope, especially when these expectations generalize across situations (Clark and Wells, 1995). In identity invalidation contexts, however, negative reactions can be more externally valid and more consequential, because legitimacy challenges are embedded in broader stigma processes and can carry concrete interpersonal, institutional, and sometimes safety-related costs (Meyer, 2003). For that reason, the present model is not intended to pathologize context-appropriate vigilance in stigmatizing environments.

The proposed pathway becomes most relevant when vigilance becomes rigid, generalized, and impairing—for example, when identity-management strategies feel mandatory across relatively safe or ambiguous contexts, self-focused attention narrows engagement, or post-event rumination amplifies future threat. At that point, the issue is not only exposure to stigma, but the maintenance of fear, self-monitoring, safety behaviors, and avoidance over time.

Accordingly, we view minority stress and social-anxiety models as complementary rather than competing accounts. Minority stress frameworks explain why invalidation-related threat is present and often realistic; the social-anxiety framework specifies one pathway through which repeated exposure may become psychologically maintained and clinically significant.

Two implications follow. First, intervention should not rely primarily on disputing threat as exaggerated; doing so may be experienced as invalidating and may ignore real-world constraints. Second, under those conditions, the clinical target becomes the maintenance loop and its generalization: helping clients (a) flexibly calibrate identity-management strategies to the situation (e.g., when to correct, disclose, disengage, or seek support), (b) reduce rigid self-monitoring and safety-behavior rules that spread beyond the original trigger, and (c) strengthen recovery after invalidation by reducing rumination and shame, reorienting attention, and returning to valued action.

## Clinical implications and possible intervention targets

The goal of this conceptual framework is not to dispute stigma or prescribe disclosure, but to suggest how invalidation may function as a trigger that recruits social-anxiety maintainers and may point to possible intervention targets for building flexibility and recovery in contexts where clients have meaningful choice and relative safety (Hendricks and Testa, 2012). The recommendations below are therefore intended as hypothesis-generating clinical implications rather than empirically established treatment procedures.

Formulation should also assess protective context (affirming relationships, supportive communities, and safer settings), because these resources can weaken the loop and help identify where practice and experiments are most appropriate. Therapeutic context should not be assumed to be affirming by default: therapist competence may help interrupt the loop, whereas subtle misrecognition, minimization of minority stress, or limited affirming competence with LGBTQ+ clients may inadvertently reproduce invalidation dynamics within treatment itself, with implications for training and supervision (Cruciani et al., 2024). The aim is not to reduce context-appropriate vigilance where risk is meaningful, but to identify when anticipation, self-monitoring, safety behaviors, and post-event processing have become overly rigid, generalized, or impairing relative to the context.

One possible formulation tool is an invalidation-triggered chain analysis. A brief map of a recent episode may help identify potential leverage points: trigger (invalidation or risk cue) → legitimacy-focused appraisal → self-monitoring → identity-management responses → post-event processing → next-situation anticipatory threat. This formulation can validate real-world risk while identifying mechanisms that may generalize beyond the original setting.

Identity management may be clinically relevant to assess as safety behavior when it functions that way. A functional assessment may help clarify in which settings concealment or appeasement is protective, and in which settings it may narrow life and maintain fear. When these strategies primarily reduce short-term anxiety in relatively safe situations, one possible therapeutic task is to explore whether they function as optional rather than mandatory responses, with attention to how much “safety” actually depends on constant management. This stance aligns with minority-stress-informed affirmative CBT approaches that acknowledge stigma while targeting proximal processes (e.g., rejection sensitivity, concealment, internalized stigma) in randomized trials with sexual minority samples (Pachankis et al., 2015, 2022).

Consent-based experiments may be one way to explore legitimacy threat and recovery. In principle, such experiments could be used to test invalidation-linked predictions (e.g., “If I correct someone, I'll be rejected”), chosen collaboratively and conducted in safer or ambiguous contexts. Potential learning targets might include tolerating ambiguity, practicing recovery after invalidation, and discovering when safety behaviors are not required to navigate or exit an interaction. Consistent with inhibitory learning approaches, one possible goal is greater flexibility under possible evaluation and learning under uncertainty, rather than certainty that negative outcomes will never occur (Craske et al., 2014).

## Research predictions

### Prediction 1 (specificity)

Legitimacy-focused invalidation predicts social anxiety beyond broad stigma exposure and beyond general internalizing. Measures indexing erasure, misrecognition, and gatekeeping should predict social anxiety severity beyond global discrimination/victimization and beyond depressive symptoms or generalized anxiety.

### Prediction 2 (mechanism)

The association between invalidation and elevated social anxiety should be partly explained by anticipatory threat appraisals, self-focused attention, identity-management safety behaviors, and post-event processing, with stigma-specific rejection sensitivity contributing to threat appraisals (Clark and Wells, 1995; Maiolatesi et al., 2023; Rapee and Heimberg, 1997).

### Prediction 3 (context and dynamics)

Context amplifies the loop, and intensive longitudinal designs reveal short-term feedback. In daily diary/EMA designs, invalidation should predict increases in identity-management behaviors and post-event processing, which should prospectively predict heightened anticipatory threat at subsequent assessments. Effects should be stronger in contexts with fewer affirming resources and higher perceived costs of visibility; a concrete proxy is living environment, where small-town/rural settings show higher social anxiety in TGNC samples and reduced buffering from social support (Kaplan et al., 2019). These dynamics may be observable dimensionally across elevated social anxiety, but should be especially consequential when they become generalized and impairing.

## Discussion

Among bi+ and nonbinary/gender-diverse people, identity invalidation is a minority stress experience that, for many, functions as a frequent and often externally valid social-evaluative threat. We propose that it can contribute to the maintenance of elevated or clinically significant social anxiety when repeated legitimacy threat recruits well-established maintenance mechanisms: anticipatory threat appraisals, identity-focused self-monitoring, identity-management safety behaviors, and post-event processing. The practical contribution is that this mechanism-focused bridge makes the problem more actionable. It suggests concrete assessment targets (legitimacy-focused invalidation, identity-management safety behaviors, and post-event processing), proposes context-calibrated directions for intervention that do not deny real risk, and generates specific tests for longitudinal and experimental work. The model also implies that treatment context matters: affirming therapist competence may help disrupt the loop, whereas invalidating care may inadvertently reinforce it. If supported, this account can refine how minority stress is translated into mechanism-level models and treatment targets for social anxiety in bi+ and nonbinary/gender-diverse populations.
